# Pfeiffer syndrome type 2 - case report

**DOI:** 10.1590/S1516-31802003000400008

**Published:** 2003-07-01

**Authors:** Maria Kiyoko Oyamada, Haideé Salgado Alonso Ferreira, Marcelo Hoff

**Keywords:** Pfeiffer syndrome, Cloverleaf skull, Craniosynostosis, Syndactyly, Upper airway, Eye, Síndrome de Pfeiffer, Crânio, Trevo, Craniosinostose, Sindactilia, Vias aéreas superiores, Olho

## Abstract

**OBJECTIVE:**

To report on a case of Pfeiffer Syndrome, with a discussion of the diagnostic characteristics and features of disease types and the differential diagnosis.

**DESCRIPTION:**

The authors describe a newborn with cloverleaf skull, extreme bilateral exorbitism and choanal atresia, partial syndactyly of the second and third toes and broad medially-deviated big toes. The case reported was Pfeiffer Syndrome type 2, which usually has a poor prognosis.

**COMMENTS:**

Pfeiffer Syndrome is a clinically variable disorder and consists of an autosomal dominantly-inherited osteochondrodysplasia with craniosynostosis. It has been divided into three types. Type 1 is commonly associated with normal intelligence and generally good outcome. Types 2 and 3 generally have severe neurological compromise, poor prognosis, early death and sporadic occurrence. Potential for prolonged useful survival outcome can be achieved in some cases with early aggressive medical and surgical management according to recent literature.

## RESUMO

**OBJETIVO:**

Apresentar relato de um caso de síndrome de Pfeiffer tipo 2, discutindo as características diagnósticas que o diferenciam dos outros tipos e os diagnósticos diferenciais.

**DESCRIÇÃO:**

Os autores descrevem recém-nascido com crânio em trevo, grave exorbitismo bilateral, atresia de coanas, sindactilia parcial cutânea de segundos e terceiros artelhos, hálux alargados e medialmente desviados, caracterizando o tipo 2 da síndrome de Pfeiffer. Esta síndrome, de manifestação clínica variável, é subdividida classicamente em três tipos distintos. O tipo 2 apresenta pior prognóstico. Chamam atenção para os recentes relatos de literatura, que reportam casos de melhor evolução, quando de abordagem clínica e cirúrgica mais agressiva.

**COMENTÁRIOS:**

A síndrome de Pfeiffer faz parte das cranioestenoses, constituindo anomalia bastante rara, com herança autossômica dominante. O tipo 1 corresponde à forma menos grave e os tipos 2 e 3 podem ser de ocorrência esporádica e apresentam anomalias muito graves.

## INTRODUCTION

In 1964, Pfeiffer described an acrocephalosyndactyly syndrome consisting of bicoronal craniosynostosis, midface deficiency, broad thumbs, broad big toes and partial and variable soft-tissue syndactyly of the hands and feet.^1^ Autosomal dominant inheritance with complete penetrance is characteristic, despite variable expressivity related to the presence or absence of syndactyly and its degree of severity.

Based on the severity of the phenotype, Cohen proposed the division of Pfeiffer syndrome (Online Mendelian Inheritance in Man classification: OMIM 101600) into 3 clinical subtypes.^2^ Classic Pfeiffer syndrome, designated Type 1, involves individuals with mild manifestations, associated with normal neurological and intellectual development, generally has good outcome and can be found dominantly inherited. Type 2 consists of cloverleaf skull, Pfeiffer hands and feet, severe exorbitism, central nervous system involvement, elbow ankylosis or synostosis. Type 3 is similar to type 2 but without the cloverleaf skull. Types 2 and 3 have poor prognosis due to severe neurological compromise and various visceral anomalies, and they generally result in early death. To date, all cases of types 2 and 3 have only had sporadic occurrence.

We report one case of Pfeiffer syndrome type 2, with particular emphasis on the clinical presentation, differential diagnosis and the importance of early diagnosis.

## CASE REPORT

**Figure 1:**
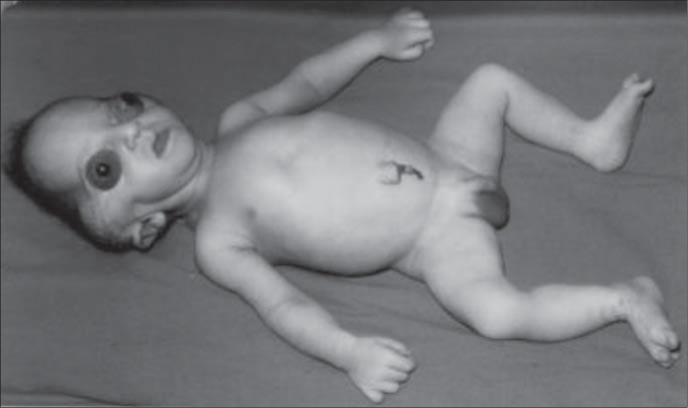
Pfeiffer syndrome type 2 - physical aspects.

**Figure 2:**
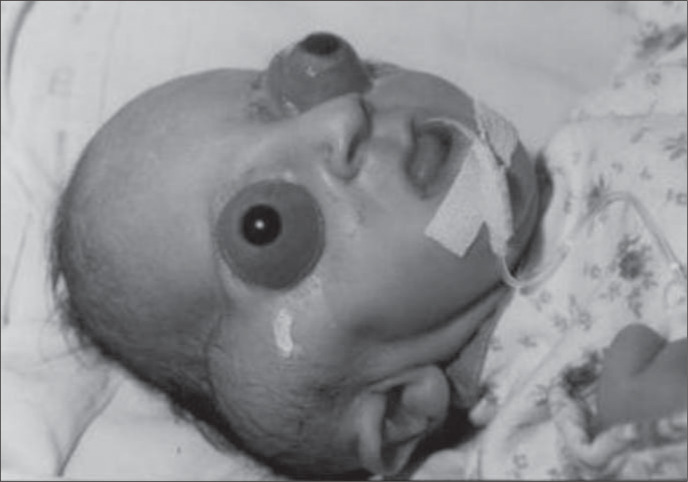
Cloverleaf skull, exorbitism and low-set ears.

**Figure 3:**
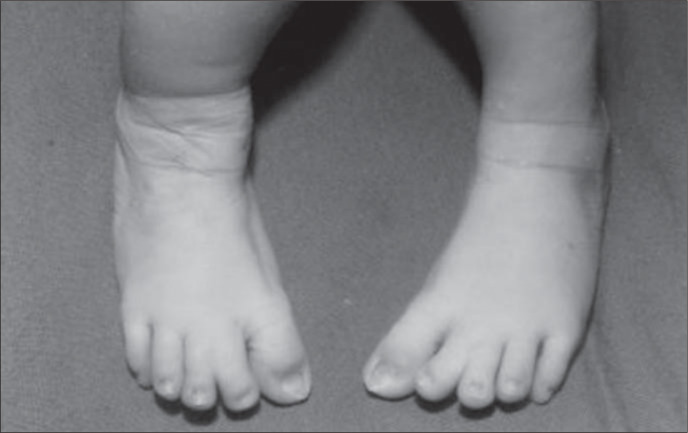
Broad big toes and cutaneous syndactyly between the second and third toes.

The patient in our case was a boy, born in 2001, as the third child of a healthy 34-yearold mother. The parents were not consanguineous, although the father was unknown. The mother had two other healthy sons from different fathers. Labor began spontaneously and the infant was delivery vaginally at term with a birth weight of 2,975 g and Apgar score of 8/9. The propositus had a cloverleaf skull, severe exorbitism, choanal atresia, low-set and posteriorly-rotated ears, broad and mediallydeviated halluces and partial cutaneous syndactyly of the second and third toes (Figures 1, 2 and 3). The ocular globes and eyelids were intact with shallow orbits that would have prevented the replacement of the eye. The ocular anterior structures were preserved, without iris or lens abnormalities. The baby developed respiratory distress and died on the second day.

**Figure 4:**
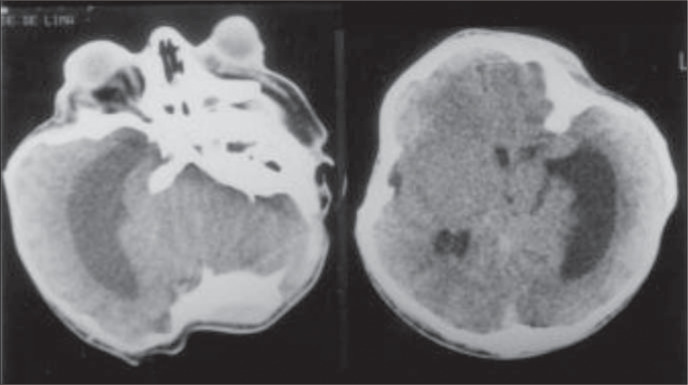
Computed tomography findings - dilatation of the lateral ventricles, cranial deformities and shallow orbit.

A computed tomography scan of the brain revealed a cloverleaf skull with cranioencephalic asymmetry, bone deformities in the cranial base and orofacial aspect, asymmetric dilatation of the lateral ventricles, cerebral abnormalities and beaked nose (Figure 4). The orbital computed tomography showed a very shallow orbit with severe proptosis and preserved optic nerve and extrinsic ocular muscles. Synostosis of the elbows was not detected on x-ray film.

## Discussion

Our patient had the clinical findings of Pffeifer Syndrome type 2 and despite this child's bad outcome, a favorable prognosis can be achieved in some cases, especially with aggressive medical and surgical intervention.^3^ For this, early clinical diagnosis is necessary.

Besides the premature craniosynostosis of coronal sutures that gives the typical appearance of cloverleaf skull deformity, midfacial hypoplasia and hand and foot anomalies, additional anomalies may also include aqueductal stenosis, hydrocephalus, cerebellar and brain stem herniation, low-set ears, external auditory canal stenosis or atresia and, unusually, hydronephrosis, pelvic kidney and hypo-plastic gallbladder.^2^

The prognostic implications and genetic counseling required are distinct and depend on the subtype. Considerable clinical overlap may occur between the three subtypes. The absence of cloverleaf skull in type 3 can result in failure to diagnose the Pfeiffer Syndrome. Presence of classic Pfeiffer hands and feet in association with craniosynostosis are the major diagnostic clues in this type of syndrome.

Although Pfeiffer Syndrome and Aperttype acrocephalosyndactyly (OMIM 101200) are noteworthy for some similarities, the two disorders appear to be nosologically and genetically distinct. Sometimes Pfeiffer Syndrome has been confused with Saethre-Chotzen (OMIM 101400) and Jackson-Weiss syndromes (OMIM 123150), since broad toes may occur in both. The big toes of Saethre-Chotzen syndrome are more triangular, with a bulbous shape and in the valgus position. Broad big toes identical to those observed in Pfeiffer Syndrome may occur in some instances of Jackson-Weiss syndrome, although broad thumbs are never observed.^2^

Congenital airway anomalies clearly related to severity of midface hypoplasia have been reported in craniosynostosis syndromes, including Pfeiffer Syndrome types 1 and 2. Upper airway anomalies are an important source of morbidity and mortality in severe Pfeiffer Syndrome, since chronic hypoventilation and hypoxia are likely to be contributory factors in neurodevelopment deficits.

The trilobed skull deformity (cloverleaf skull) is a rare congenital anomaly that may be present as an isolated defect, but is usually part of an osteochondrodysplastic or dysostotic syndrome like Apert, Crouzon (OMIM 123500) or Pfeiffer syndrome. This anomaly has important prognostic implications because of the limited brain growth and the eye exposure caused by shallow orbits. The pathogenesis of the cloverleaf skull is unknown, but the trilobed shape of the head, hydrocephalus and facial deformation have been attributed to intrauterine synostosis of cranial sutures.^3^ Abnormalities of the cranial base may have played a role in the respiratory distress of the baby reported.

The ocular manifestations in Pfeiffer Syndrome may be an inherent feature of the pathological process or may occur as a secondary complication. They can include hypertelorism, downslanting palpebral fissures, strabismus, anterior chamber dysgenesis including Peter's anomaly (corneal clouding and variable iridolenticulocorneal adhesions), corneal scleralization, corectopia, atypical iris colobomata superiorly, retinal detachment and atrophic optic nerve heads. Disc edema, optic atrophy, and progressive optic nerve dysfunction may accompany increased intracranial pressure even without evidence of hydrocephalus. Severe proptosis may cause corneal ulcer, endophthalmitis and globe rupture.

Experience with corrective surgery is limited. The aims of the surgery are: decompression of the brain and remodeling of the skull, elongation and expansion of the bony orbits to accommodate the globes and enable eyelid closure, and opening of the nasopharyngeal airways by advancement of the naso-maxillary-zygomatic complex.^3^

Recent molecular genetic research has done much to advance our understanding of the molecular basis for some craniosynostosis syndromes. Pfeiffer Syndrome, either in familial or sporadic cases, has been associated with fibroblast growth factor receptor 1 and 2 (FGFR1 locus 8p11.2-p11.1, OMIM 136350; and FGFR2 locus 10q26, OMIM 176943) gene mutations.^4,5^ Severe midface hypoplasia and exorbitism are more likely to be associated with FGFR2 gene mutation. Identical mutations in the fibroblast growth factor receptor gene have been identified in Crouzon, Jackson-Weiss and Pfeiffer Syndrome types 2 and 3, thereby resulting in variable expression with distinct phenotypes.^4,6^ It has been demonstrated that the FGFR2 gene is also involved in the development of the anterior chamber of the eye. Ser351Cys mutation has been identified in craniosynostosis patients with Peter's anomaly and other anterior ocular chamber defects, and is likely to be associated with a severe phenotype and clinical course.^7^ Sporadic cases have been related to advanced paternal age, and there has been speculation that older men are more susceptible to a variety of germline mutations.^8^

The more severe types of Pfeiffer Syndrome are due only to *de novo* mutations, but it is not possible to rule out the presence of mosaicism in one of the parents. Familial recurrence risk may be considered within the scope of genetic counseling.

Prenatal diagnosis can occasionally be made with three-dimensional ultrasound based on ultrasound findings like head shape, facial hand and foot abnormalities.^9^ Unfortunately, clinical management has major problems and experience with corrective surgery is very limited, but the potential for a favorable outcome may be greater with prenatal diagnosis.

### Electronic-Database Information

Online Mendelian Inheritance in Man (OMIM), available at URL http://www.ncbi.nlm.nih.gov/Omim/
